# Characterization of Structural Bone Properties through Portable Single-Sided NMR Devices: State of the Art and Future Perspectives

**DOI:** 10.3390/ijms22147318

**Published:** 2021-07-07

**Authors:** Marco Barbieri, Paola Fantazzini, Claudia Testa, Villiam Bortolotti, Fabio Baruffaldi, Feliks Kogan, Leonardo Brizi

**Affiliations:** 1 Department of Radiology, Stanford University, Stanford, CA 94395, USA; fkogan@stanford.edu; 2 Department of Physics and Astronomy “Augusto Righi”, University of Bologna, 40127 Bologna, Italy; paola.fantazzini@unibo.it (P.F.); claudia.testa@unibo.it (C.T.); 3 IRCCS Istituto delle Scienze Neurologiche Bologna, Functional and Molecular Neuroimaging Unit, 40139 Bologna, Italy; 4 Department of Civil, Chemical, Environmental, and Materials Engineering, University of Bologna, 40134 Bologna, Italy; villiam.bortolotti@unibo.it; 5 Medical Technology Laboratory, IRCCS Istituto Ortopedico Rizzoli, 40136 Bologna, Italy; fabio.baruffaldi@ior.it

**Keywords:** bone, single-sided NMR, NMR relaxometry, osteoporosis, structural parameters

## Abstract

Nuclear Magnetic Resonance (NMR) is a well-suited methodology to study bone composition and structural properties. This is because the NMR parameters, such as the $T_{2}$ relaxation time, are sensitive to the chemical and physical environment of the ^1^H nuclei. Although magnetic resonance imaging (MRI) allows bone structure assessment in vivo, its cost limits the suitability of conventional MRI for routine bone screening. With difficulty accessing clinically suitable exams, the diagnosis of bone diseases, such as osteoporosis, and the associated fracture risk estimation is based on the assessment of bone mineral density (BMD), obtained by the dual-energy X-ray absorptiometry (DXA). However, integrating the information about the structure of the bone with the bone mineral density has been shown to improve fracture risk estimation related to osteoporosis. Portable NMR, based on low-field single-sided NMR devices, is a promising and appealing approach to assess NMR properties of biological tissues with the aim of medical applications. Since these scanners detect the signal from a sensitive volume external to the magnet, they can be used to perform NMR measurement without the need to fit a sample inside a bore of a magnet, allowing, in principle, in vivo application. Techniques based on NMR single-sided devices have the potential to provide a high impact on the clinical routine because of low purchasing and running costs and low maintenance of such scanners. In this review, the development of new methodologies to investigate structural properties of trabecular bone exploiting single-sided NMR devices is reviewed, and current limitations and future perspectives are discussed.

## 1. Introduction

Osteoporosis is a disease associated with increased bone fragility and susceptibility to fractures, which poses a high economic burden on health systems and has a severe impact on the quality of human health [1,2,3]. People experiencing an osteoporotic fracture are exposed to various adverse outcomes in terms of morbidity, subsequent fractures, and mortality after hospital discharge [4]. Methodologies aiming to improve early and accurate assessment of the fracture risk related to osteoporosis are necessary to effectively treat osteoporotic patients to prevent the worsening of bone health before a fragility fracture occurs. Wide campaigns of screening the population at risk of osteoporosis are desirable, which motivates the development of techniques that are low-cost and easily accessible to allow routine application.

Dual-energy X-ray absorptiometry (DXA) is currently the most commonly utilized clinical tool for the diagnosis of osteoporosis [5]. It employs ionizing radiation and provides a measure of the areal bone mineral density (BMD), but it does not provide information about the architecture of the bone [3,6]. Other than BMD, bone strength depends on the size, shape, architecture, and composition of the tissue [5]. For example, the resilience of the trabecular bone depends not only on BMD but also on the architecture of the trabecular structure. Furthermore, epidemiological studies showed that BMD is a poor predictor of fracture risk, the low values of BMD being able to explain about 50% of the incident fracture cases [7]. Models for risk fracture evaluation that integrate BMD with anthropometric factors, lifestyle factors, and comorbidities have been proposed and shown to increase fracture risk prediction accuracy [8,9]. Despite the evidence that bone strength, and by consequence the fracture risk, also depends on bone architecture and composition, there are no well-studied models that incorporate information about bone architecture or composition in fracture risk assessment. This is largely due to the absence of an exam able to provide such information that is suitable for routine clinical practice.

Although high-resolution quantitative Computed Tomography (HRqCT) can provide volumetric 3D measurements of cortical and trabecular bone in vivo, it requires a higher X-ray dose than DXA, is more expensive, and is primarily limited to peripheral skeleton bones. Therefore, the use of these scans in a clinical setting is infrequent [5]. Quantitative ultrasounds (qUS) have also been proposed as prescreening methodology for study bone mineralization and some structural properties [10] and have the advantage of being low-cost and do not use ionizing radiation. Although qUS-based techniques for bone assessment have been around since the early 1990s [10], measurements are still affected by low repeatability and reproducibility [11,12].

Nuclear Magnetic Resonance (NMR) is a well-suited technology to study bone structural properties and its composition. It does not use ionizing radiation, and laboratory studies have shown the ability of NMR to assess properties of bone with different techniques, ranging from relaxometry to diffusometry and Magic Angle Spinning [13,14,15,16,17,18,19,20,21,22]. Although clinical magnetic resonance imaging (MRI) allows bone structure assessment in vivo [23,24,25], its cost limits the suitability of conventional MRI for routine bone screening. The use of more compact and lower magnetic field scanners has been proposed to lower the cost of MRI analyses for imaging trabecular bone structure on peripheral body regions [26,27]. Furthermore, bone is a complex tissue, and assessment of bone properties with quantitative MRI in acquisition times suitable for clinical application is challenged regarding the need of spatially encoding the NMR signal.

Portable NMR, based on low-field single-sided NMR devices, is a promising and appealing approach to assess NMR properties of biological tissues, particularly for relaxometry and diffusometry, with the aim of medical applications. The main feature that differentiates a single-sided apparatus from other NMR scanners is the detection of the signal from a sensitive volume external to the magnet. Therefore, such devices can be used to perform NMR measurement without the need to fit a sample inside a bore of a magnet, allowing, in principle, in vivo application not limited to peripheral body sites. Moreover, the intrinsic magnetic field gradients can be used for spatial encoding. Portable NMR also has the added advantages of low purchasing, running, and maintenance costs, largely due to the absence of superconducting magnets (permanent magnets are employed instead). Portable NMR devices [28,29,30,31,32,33,34] have been applied in various fields such as agriculture [35], cultural heritage [36,37,38], porous media [39], and biomedicine including testing of silicone breast implants [40] and ex vivo studies of various biological tissues: tendon [41], articular cartilage [42,43], skin [44], breast [45], and bone tissue [46,47,48,49,50]. In vivo studies on humans have also been conducted on skin [51] and tendons [52].

In this review, the development of new methodologies to investigate trabecular bone properties in laboratory exploiting single-sided NMR devices is reviewed, and current limitations and future perspectives are discussed. In Section 2, a brief background on NMR relaxometry of the bone tissue is provided. A few details about single-sided NMR devices are given in Section 3. Works investigating the characterization of trabecular bone structure using single-sided NMR are reviewed in Section 4. In Section 5, current limitations and future perspectives are discussed with a specific attention to the long-term aim, i.e., the extension of such techniques to an in vivo application.

## 2. NMR Relaxometry Properties of Bone Tissue

### 2.1. Bone Tissue and Osteoporosis: A Brief Background

Bone is a complex composite including type-I collagen, calcium phosphate mineral deposits, lipids, and water confined in a network of nano-, micro-, and macrostructural compartments. Bone can be classified in cortical bone (CB) and trabecular bone (TB), characterized by different structures. The components of CB are arranged in a porous space made of the Haversian canals and the lacunar-canalicular system, which occur in repeating cylindrical units called osteons. TB, predominant in the axial skeleton and near the joints of the long bones, consists of a network of interconnected trabeculae, typically 100–150 μm thick, where the lacunar-canalicular porosity and the collagen–apatite porosity is found, as for the cortical bone. In TB, there is further porosity contributed by the inter-trabecular space containing marrow, fat, and blood vessels, but not the Haversian canals.

The bone tissue experiences different loads in intensity and directions over life-time, which causes an adaptive response of the bone tissue, such as altering structural density and orientation (i.e., creating anisotropy) [53]. This adaptive response is possible because of bone remodeling, a process where bone tissue is removed by osteoclastic resorption and new bone is formed by osteoblasts. In the adult skeleton, the bone tissue is remodeled in a process that has a dynamic equilibrium between bone formation and bone resorption. With aging and in the context of osteoporosis, this equilibrium is broken, and the balance of bone resorption and formation becomes negative. Thus, the bone loss in aged and osteoporotic bone is a consequence of imbalanced and excessive bone remodeling [54], which increases fracture risk.

Since bone remodeling occurs on the surfaces of internal bone structures, osteoporotic bone loss is a function of the surface available for bone remodeling [55]. In individuals, less than 65 years of age, the trabeculae of the trabecular bone furnish the largest surface available for bone remodeling. In this population, trabecular bone provides only about 20% of the skeletal bone mass, but it is responsible for most of the turnover [53]. Thus, the bone loss in early osteoporosis is mainly due to trabecular bone [53]. With the advance of the disease, the porosity of the cortical bone increases as well, and, therefore, its endocortical surface increases. As a consequence, the largest loss of absolute bone mass due to osteoporosis in an advanced state occurs in cortical bone by intracortical remodeling [53]. These considerations allow one to appreciate the importance of assessing bone structural properties, and the importance of having techniques able to conveniently gain that information to allow early detection of osteoporotic behavior of the bone tissue. Some of the most common parameters for structure characterization of trabecular bone are:

- BONE VOLUME/TOTAL VOLUME, BV/TV (%): % of total volume of the trabeculae with respect to the total volume of the analyzed tissue;
- BONE SURFACE/TOTAL VOLUME, BS/TV (mm^−1^): total external surface of the trabeculae with respect to the total volume of the analyzed tissue;
- TRABECULAR THICKNESS, Tb.Th (mm): the mean trabecular thickness found in the trabeculae of the analyzed tissue.

### 2.2. NMR Relaxation in Porous Media and Bone Tissue

In this section, the NMR signal of ^1^H nuclei, usually called proton NMR, is considered. NMR relaxation parameters, such as $T_{1}$ and $T_{2}$ relaxation times, both depend on the chemical and physical environment of the ^1^H nuclei and can be used to characterize these environments. The information provided by NMR relaxometry has constituted a powerful non-invasive and non-destructive tool for studying the structural properties of porous media [14,56,57].

In biological tissues, ^1^H nuclei can be present in different chemical environments (water or fat, for example), and some tissues, from an NMR perspective, can be modeled as porous media in which fluids are confined in complex porous structures. Thus, a multi-exponential relaxation process is often encountered. The acquired multi-exponential decay data can be analyzed by quasi-continuous $T_{1}$ and $T_{2}$ distributions, able to give unique information about the tissue under study. In the context of bones, the ^1^H nuclei are found in different micro-environments that are too small to be spatially resolved with MRI, but their NMR signal contributions can be resolved in the time domain using the multi-exponential NMR relaxation approach.

NMR relaxometry investigations on cortical and trabecular bones have been extensively studied in the literature, and many studies have shown that $T_{2}$ relaxation times span a range that goes from tens of µs to hundreds of ms [14,15,25,49,58,59]. Hence, the different $T_{2}$ components can be mapped to the corresponding ^1^H nuclei micro-environments.

**Figure 1:**
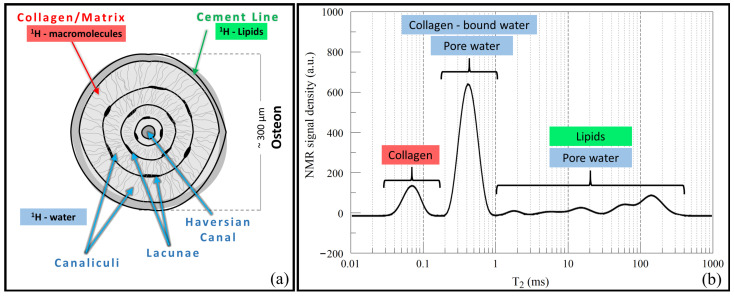
(**a**) Schematic representation of cortical bone indicating its constituents and compounds from which the ^1^H NMR signal arises; (**b**) $T_{2}$ distribution of human cortical bone (plot from data presented in reference [58]).

Figure 1 summarizes the $T_{2}$ relaxation properties of human cortical bones found by Horch et al. [58,59] using quasi-continuous $T_{2}$ distributions, obtained by an inversion process of the Carr–Purcell–Meiboom–Gill (CPMG) measured data [60,61]. The $T_{2}$ distributions (Figure 1b) present two distinct sub-millisecond relaxation components, and a broad tail spanning the millisecond-second $T_{2}$ domain. The first sub-millisecond component ($T_{2}∼60\;\mu s$) has been attributed to the collagen methylene protons, while the second sub-millisecond component ($T_{2}∼400\;\mu s$) contains signal contributions from collagen-bound water and water within the lacunar-canicular system (order 0.1 μm [62]). The long-lived tail spanning the millisecond-second domain contains signal contributions from water within the Haversian canals and lipids present within the cement lines (regions of collagen-poor bone matrix found at the outer border of osteons). It is important to note that the signal contributions due to water within Haversian canals and the lacunar-canicular system is generally referred to as pore water. However, the spatial scale of pores within cortical bone varies considerably and thus the $T_{2}$ relaxation times vary accordingly to the size of the pore structure.

**Figure 2:**
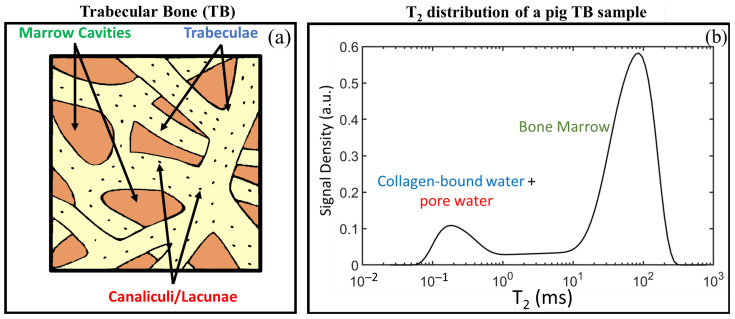
(**a**) Schematic representation of trabecular bone indicating its constituents; (**b**) $T_{2}$ distribution of a trabecular bone sample cored from a pig shoulder (plot from data used in reference [50]).

Fantazzini et al. [14] conducted NMR relaxometry studies on animal trabecular bones finding that trabecular bone $T_{2}$ distributions present a distinct sub-millisecond relaxation component and a broad component spanning the millisecond-second domain that accounts for most of the signal. The first sub-millisecond component ($T_{2}∼400\;\mu s$) was attributed to signals coming from protons within the trabeculae (intra-trabecular signal), which includes collagen-bound water and pore water. The second component was attributed to fluids within the inter-trabecular spaces. Figure 2 presents a schematic representation of trabecular bone along with a $T_{2}$ distribution obtained from a trabecular bone sample cored from a pig shoulder [50] to highlight the connection between the $T_{2}$ components and the trabecular bone constituents.

It is important to notice that MRI has been incorporating the concepts of NMR relaxometry in quantitative MRI (qMRI) methodologies such as $T_{1}$ and $T_{2}$ mapping. However, these methodologies are challenged by technical limitations that arise from the constraints imposed by the need to spatially encode the NMR signal. For these reasons, the majority of qMRI protocols that can be run in clinically acceptable times apply mono-exponential models to processes that often are multi-exponential.

## 3. Single-Sided NMR Devices

Single-sided NMR devices detect the signal from a sensitive volume positioned above the surface of the magnet and the radiofrequency (RF) coil, which implies that there are no strict requirements about the size of the sample to be investigated. They use permanent magnets to generate the polarizing field $B_{0}$, usually on the order of hundreds of mT, with a significantly small size compared to high-field NMR and whole-body MRI scanners. Altogether, these characteristics make single-sided NMR scanners portable, low maintenance, low purchasing costs, and suitable for in situ measurements.

**Figure 3:**
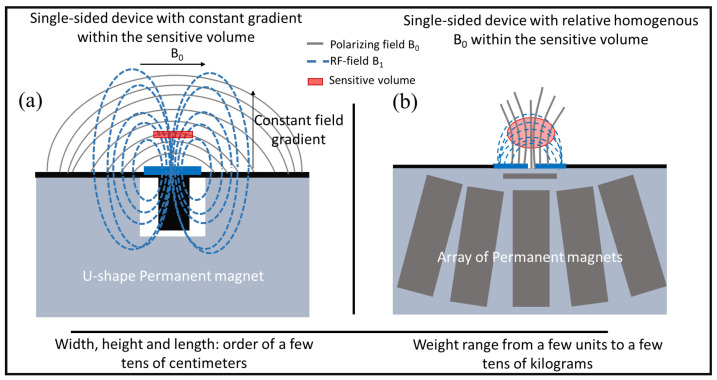
(**a**) Sketch of the design of a single-sided NMR device with a constant field gradient within the sensitive volume; (**b**) sketch of the design of a single-sided NMR device with a relative homogeneous $B_{0}$ within the sensitive volume.

Different magnet geometries have been explored for single-sided NMR [28,29,30,31,32,33,34]. The main difference among these scanners is the design and disposition of the permanent magnets, which determines the polarizing magnetic field and consequently the position and shape of the sensitive volume, the volume where the ^1^H nuclei will be excited by a suitable RF pulse. Two main classes can be differentiated, taking into account the distribution of the static magnetic field. In the first class [28,30,31,32], one or more magnets are used to generate a grossly inhomogeneous $B_{0}$ field, and an RF coil is designed such that the excitation field $B_{1}$ and the static field $B_{0}$ are orthogonal within a specific region above the surface of the magnet. The $B_{0}$ gradient and the excitation bandwidth define the sensitive volume. A sub-class of such devices generates a uniform constant $B_{0}$ gradient within the sensitive volume, allowing for diffusivity measurements [28,32,33]. The second class of instruments [29,31,34] generates a *sweet spot* at which $B_{0}$ is locally homogeneous. Figure 3 presents a schematic representation of two single-sided NMR devices belonging to the two classes described above. Until recently, the maximum penetration depth achieved by portable single-sided NMR devices ranged from a few mm to 2 cm. Thomas et al. [34] presented a novel single-sided NMR device with a maximum penetration depth of 50 mm.

## 4. Evaluating Trabecular Bone Structural Properties of Animal Samples with Single-Sided NMR

**Figure 4:**
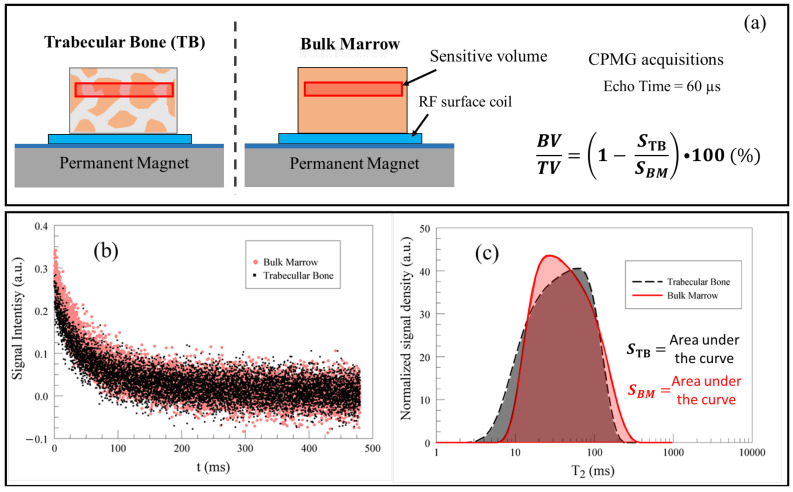
Evaluation of BV/TV ratio of trabecular bone in [47]. (**a**) Sketch of the experimental set-up; (**b**,**c**) pipeline used to evaluate the BV/TV ratio of trabecular bone; (**b**) signal intensity decays; (**c**) $T_{2}$ relaxation time distribution obtained by UpenWin software, implementing the Upen algorithm [63]. Since the experimental set was not optimized for SNR efficiency, the sub-millisecond decay component ascribed to the intra-trabecular bound, and pore water was not detected in the $T_{2}$ distribution so that it did not contribute to the signal.

A procedure to assess the bone volume fraction of trabecular bone using single-sided NMR devices was first proposed in [47] in the laboratory using ex vivo animal samples. The procedure, whose pipeline is summarized in Figure 4, is based on computing the ratio between the CPMG signals obtained from a trabecular bone sample and the CPMG signal obtained from a reference bulk marrow sample. As long as the volumes of the samples intercepted by the sensitive volume of the single-sided NMR scanner are the same, the ratio of the two signals (where the signal is the total area under the $T_{2}$ distribution computed out of the CPMG decay) is proportional to the ratio between the volume of the bone marrow within the inter-trabecular spaces and the total volume of the sample. The bone volume fraction can then be evaluated according to Equation (1), where $S_{TB}$ is the NMR signal from the marrow inside the trabecular bone sample, and $S_{BM}$ is the NMR signal from a reference bulk marrow sample:

(1) $$\frac{BV}{TV}=\left(1-\frac{S_{TB}}{S_{BM}}\right)\times 100\left(\%\right)$$

**Figure 5:**
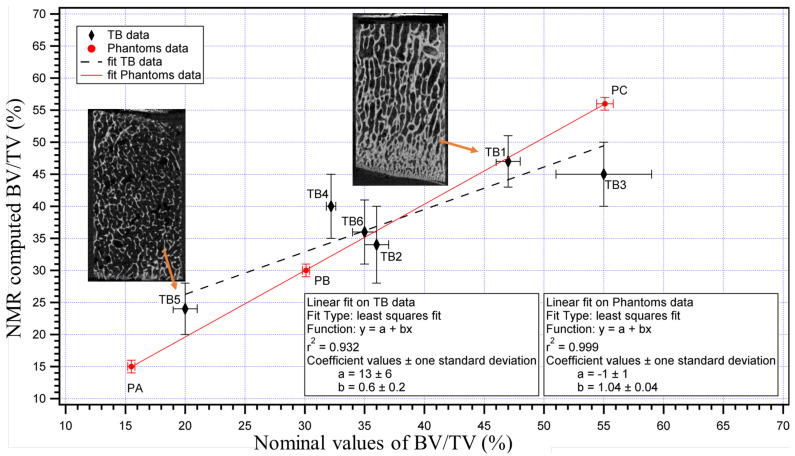
BV/TV values of TB animal samples and phantoms samples measured by single-sided scanner NMR-MOUSE PM10 (Magritek, Nz) vs. the nominal values. For the TB samples, the nominal values are the BV/TV values determined by the micro-CT analysis, whereas, for the phantoms, they have been obtained by the geometry of the samples. Examples of micro-CT images are reported for two TB samples. The figure was modified from reference [47]. Copyright (2017) Wiley, Copyright (Year) Wiley. Used with permission from reference [47].

The methodology was validated on a set of phantoms of which ground truth BV/TV could be accurately computed by their geometry and on a set of six trabecular bone samples where the micro-CT was used to estimate the reference BV/TV. The validation experiments presented in [47], reported in Figure 5, showed a high correlation between phantoms and trabecular bone samples.

**Figure 6:**
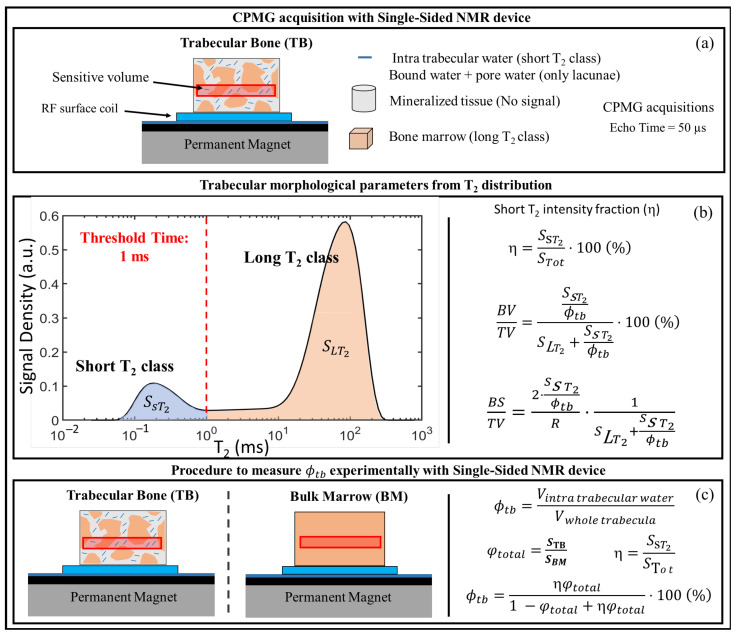
Sketch of the pipeline used in laboratory to evaluate the structural parameters of animal trabecular bone samples in [50]. (**a**) Experimental set-up; (**b**) separation on the $T_{2}$ distribution of the signal from ^1^H inside the trabeculae from that coming from the marrow within the inter-trabecular spaces, through the definition of a threshold time, set at 1 ms. On the right, the theoretical relationships to estimate the two parameters BV/TV and BS/TS are summarized, starting from the computation of η, i.e., the ratio of the short $T_{2}$ signal to the total signal under the $T_{2}$ distribution. R is the average radius of the trabeculae, estimated from the average trabecular thickness, measured with micro-CT; (**c**) experimental procedure to measure the total porosity and the intra-trabecular porosity.

A limitation of the procedure presented in [47] is the need to use a reference sample to compute the BV/TV ratio. If the volume of the reference sample differs from the volume of the trabecular bone intercepted by the sensitive volume of the single-sided NMR scanner, the evaluation of BV/TV is no longer accurate. Moreover, the need for an external reference could be a drawback in a possible future in vivo application. The procedure was then further developed in [50], reducing at least in part the need for a reference sample and extending the study to the estimation of the structural parameter BS/TV (% of the total external surface of the trabeculae with respect to the total volume of the analyzed tissue). These goals were achieved by optimizing the experimental set-up to maximize the Signal-to-Noise ratio, giving a physical interpretation to the $T_{2}$ quasi-continuous distributions, and by applying simple theoretical models to obtain NMR estimates of BV/TV and BS/TV. The NMR estimates obtained in laboratory using animal samples were validated by the comparison with the values of these parameters measured by micro-CT analyses. Figure 6 summarizes the pipeline used to implement the methodology presented in [50] along with the theoretical models used to estimate BV/TV, BS/TV, and the intra-trabecular porosity, $\phi_{tb}$. The latter parameter was first introduced in [15] and is defined as the volume within the trabeculae occupied by the fluids divided by the total volume of the trabeculae. The reader is invited to read reference [50] for a detailed derivation of the models.

**Figure 7:**
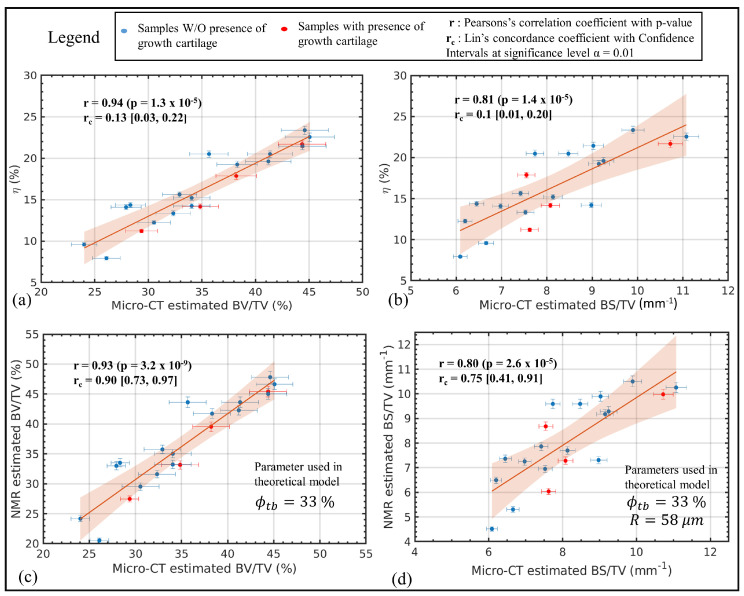
(**top row**) Plots of η, the short $T_{2}$ intensity fraction, evaluated using the quasi-continuous $T_{2}$ distributions, against the structural parameters evaluated by the micro-CT analyses. (**a**) the BV/TV; (**b**) the BS/TV (**bottom row**) Comparison between NMR and micro-CT estimated structural parameters; (**c**) BV/TV; (**d**) BS/TV. The average intra-trabecular porosity $\phi_{tb}$ = 33%, measured experimentally, was used in the theoretical models to estimate BV/TV and BS/TV with NMR. For the BS/TV, the trabeculae were modeled as cylinders and the the average radius R = 58 μm, estimated from average trabecular thickness measured with micro-CT, was used. The figure was modified from reference [50]. Copyright (2020) Wiley. Used with permission from reference [50].

The key idea was to exploit the information contained in the $T_{2}$ distribution of the trabecular bone sample computed from the acquired CPMG data to separate the intra-trabecular signal (due to water bound to collagen and pore water) and the signal coming from the inter-trabecular spaces (mainly due to bone marrow). This allowed one to compute the short $T_{2}$ intensity fraction, namely η, proportional to the volume occupied by fluids inside the trabeculae to the volume occupied by all fluids in the bone, including fluids within the spaces between the trabeculae. This parameter was found to strongly correlate with BV/TV and with the so-called bone surface density BS/TV, as shown in Figure 7a,b. Furthermore, the correlation between NMR and micro-CT estimated structural parameters showed a high level of agreement for BV/TV and a high correlation with a moderate agreement for BS/TV as summarized in Figure 7c,d, respectively. A further novelty of the work was demonstrating the possibility of quantifying with a single-sided NMR device the intra-trabecular porosity (see Figure 6c), a parameter not easy to measure by other methods. The study concluded that, for trabecular bones cored from the shoulder of healthy pigs, the average intra-trabecular porosity, $\phi_{tb}$, was equal to (30 ± 5)%. The value of the intra-trabecular porosity was almost constant regardless of the BV/TV of the samples, which spanned the range (24–45)%.

## 5. Discussion: Current Limitations and Future Perspectives

Obtaining information about the trabecular bone structure in addition to the BMD has been demonstrated to improve the fracture risk prediction associated with the osteoporosis disease. Methodologies able to gain this information in vivo, such as MRI-based methodologies, are not currently part of the clinical routine because of the high financial cost associated with MRI exams. NMR relaxometry has been demonstrated to be a well-suited methodology to study bone composition and structure. The ^1^H nuclei present in the bone are found in different micro-environments, and their NMR signal contribution can be resolved in the $T_{2}$ quasi-continuous distributions. The use of portable single-sided NMR devices for biomedical applications is an emerging and appealing field because of their versatility and low purchasing and running costs. In this work, the state of the art of new methodologies that aimed to investigate trabecular bone structural properties exploiting a single-sided NMR device has been reviewed, highlighting the concepts behind the methods rooted in NMR relaxometry applied to the study of porous media.

The methodologies reviewed in this work showed that portable single-sided NMR devices could be efficiently utilized to retrieve structural information of trabecular bones in a laboratory study. A novelty of [50] was the demonstration of the possibility to quantify the intra-trabecular porosity, a parameter not easy to measure by other methods. The literature does not establish the extent to which diseases affecting bone tissue, such as osteoporosis, modify its structure at the intra-trabecular level. It is not clear whether the imbalanced and excessive bone remodeling that occurs in bone pathologies only affects the trabecular network, by removing some trabeculae, or whether it also affects the internal porosity of the trabeculae themselves. Hence, the technique can readily be used to investigate these effects in laboratory studies exploiting a non-destructive methodology that uses low-cost portable single-sided NMR devices.

The long-term aim of these procedures is in vivo application for broad screening campaigns, but the methodology is still far from that goal. Some current limitations and challenges have to be addressed to move the technique forward. The main challenges related to an in vivo application of these techniques regard the correct positioning of the sensitive volume of the single-sided scanner within the chosen anatomical site. Commercially available single-sided NMR scanners have a maximum penetration depth of about 20 mm. With the envisage of application in humans, the limited achievable penetration depth would restrict the application of the technique to peripheral skeletal regions. In these anatomical sites, tissues other than trabecular bone, such as muscles or cartilage, may be present within the sensitive volume of the scanner affecting the correct estimation of structural parameters. A possible methodological solution is to explore the modification of the reviewed techniques to select only the signal coming from trabecular bone tissue by filtering out signal contributions from tissues surrounding the bone as investigated in [48]. In that work, the procedure proposed in [47] was modified using a suitable preparatory pulse sequence able to filter the signal in diffusion before the CPMG acquisition. This means to cancel out the signal from higher diffusion coefficient molecules before acquisition. The proposed modification correctly estimated the BV/TV of a trabecular bone sample even when muscle and cartilage tissues were present within the sensitive volume of the single-sided scanner. This was possible because water molecules in muscle and cartilage have diffusion coefficients about two orders of magnitude higher than those of fluids in bone marrow (mainly lipids). The concept of a diffusion filter, as proposed in [48], would not be straightforwardly applicable to the procedure developed in [50]. The diffusion preparation before the CPMG acquisition would also filter out the sub-millisecond $T_{2}$ components, components assigned to the water inside the trabeculae, which would eliminate the internal reference signal. New approaches that aim to integrate the concept of a diffusion filter without losing the information coming from the intra-trabecular water need to be further explored.

Another approach to face the challenges related to the positioning of the sensitive volume is to design an ad-hoc single-sided scanner for a specific bone district of interest. Thomas et al. [34,64] presented a novel single-sided NMR device with a maximum penetration depth of 50 mm, which was then successfully used to study brain hypoxia in an ovine model in vivo. These recent studies show that technological advances in the design of single-sided NMR scanners to reach the desired properties in terms of sensitive volume size, shape, and penetration depth are not just a matter of mere speculation. A single-sided NMR scanner with a penetration depth of a few cm allows for envisioning an application of the techniques reviewed here that is not only restricted to peripheral bones but could potentially be applied to other body regions of interest, such as the hip. Furthermore, augmented reality approaches can be explored to improve the proper positioning of the sensitive volume. Augmented and mixed reality frameworks have increased interest in the last years by combining 3D MRI or CT imaging with holographic vision devices for surgical planning [65,66,67,68,69]. However, these approaches require a 3D image of the anatomical site of interest, which is usually done through MRI or CT acquisitions. In the context of surgery planning, a 3D imaging modality is already necessary and part of the standard clinical routine. On the contrary, in the context of osteoporosis assessment, 3D imaging is not routinely applied. However, since recent works have shown the feasibility of rendering 3D bone shapes from 2D DXA scans [70,71], one can envision exploiting these methodologies as anatomical information to be combined with the augmented reality framework to improve the proper positioning of the sensitive volume of single-sided NMR devices within the anatomical site of interest. DXA scans are already routinely applied to assess bone health so that no additional financial costs and time would be required.

Lastly, the specific absorption rate (SAR) associated with the procedures reviewed in this work has not been studied. SAR measures the amount of radiofrequency power absorbed per unit of mass of a tissue and needs to remain below specific levels to guarantee that a procedure does not cause excessive heating, resulting in burn injuries to patients. Measuring SAR is not trivial, but it scales with the square of the RF power. The methodology presented in [50] utilizes a CPMG with a train of 2000 refocusing pulses. Reducing the number of refocusing pulses will also reduce the RF power deposited in the tissue. The use of the log-spaced CPMG sequences [72,73], where the time delay between consecutive refocusing pulses is logarithmically spaced instead of being kept fixed, can be explored to reduce the number of pulses needed to sample the CPMG decay. It has been demonstrated that log-CPMG can accurately reproduce the $T_{2}$ distributions of rock samples where $T_{2}$ spanned the sub-millisecond–hundreds of milliseconds range [73]. Thus, log-CPMG may be a valuable tool to accommodate the SAR requirements that need to be met to apply the methodology in vivo.

In conclusion, this review gives grounds for confidence in the usefulness of single-sided NMR for studying bone porosity at different scale levels. The available techniques are considered mature for ex vivo preclinical investigation of the relationship between bone diseases, such as osteoporosis, and the structure of the bone, even at the intra-trabecular level. The methodology is still a long way from possible extension to an in vivo application. However, the challenges associated with this long-term aim have been discussed, and possible solutions to tackle each of these challenges have been proposed starting from readily available technology. This suggests that it is realistic to envisage future screening campaigns conducted on populations at risk of bone diseases, such as osteoporosis, using low-cost and portable single-sided NMR devices [74,75]. 

## Abbreviations

The following abbreviations are used in this manuscript:

BS/TV	Bone-surface to total-volume ratio
BV/TV	Bone-volume to total-volume ratio
CB	Cortical Bone
CPMG	Carr–Purcell–Meiboom–Gill
CT	Computed Tomography
HRqCT	High-Resolution quantitative Computed Tomography
MRI	Magnetic Resonance Imaging
NMR	Nuclear Magnetic Resonance
qMRI	quantitative Magnetic Resonance Imaging
qUS	quantitative Ultrasounds
TB	Trabecular Bone
Tb.Th	Trabecular Thickness
